# Microchamber Extraction and Analytical Pyrolysis to Explore Volatile Organic Compounds from Compression-Cooking Wood Materials Obtained under Different Conditions

**DOI:** 10.3390/molecules27238260

**Published:** 2022-11-26

**Authors:** Elise Bertheau, Valérie Simon, Christine Delgado Raynaud

**Affiliations:** 1 Laboratoire de Chimie Agro-Industrielle, LCA, Université de Toulouse, INRAe, 4 allée Emile Monso, 31030 Toulouse, France; 2 Centre d’Application et de Traitement des Agro-Ressources (CATAR), Toulouse-INP, 4 allée Emile Monso, 31030 Toulouse, France

**Keywords:** volatile organic compounds, emissions, heartwood, *Quercus robur*, microchamber, pyrolysis

## Abstract

Volatile organic compounds are species of concern for indoor air quality. They are emitted from a wide range of indoor sources and in particular from construction materials. Industrialized wood-based panels made from various types of wood bonded with thermosetting adhesive resins have been shown to emit volatile organic compounds over months or even years mostly due to the petrochemical binders. Some studies have been conducted on binderless panels, but they mainly focused on the pressing parameters to be applied to optimize the panel characteristics. The aim of this research is to document the emissions from binderless panels and to access the volatile composition of wood processing through the molding of materials. For this purpose, binderless boards were manufactured from hardwoods, known to emit less than softwoods with different thermopressing temperatures and times. Emissions were studied by placing the materials in microchambers. Volatile organic compounds were then sampled and analyzed by various chromatographic methods. On the other hand, materials were pyrolyzed and then analyzed by gas chromatography and mass spectrometry. The implemented protocols proved suitable for the determination of more than 40 organic compounds, among which are aldehydes, aromatics, furans and derivatives, and carboxylic acids.

## 1. Introduction

Volatile organic compounds (VOCs) are defined by the World Health Organization (WHO, Geneva, Switzerland) as compounds with a boiling point of 50–250 °C at an air pressure of 101.32 kPa [1]. They have received much attention from researchers who have studied their ecological role, their toxicity, and their impact on photochemical pollution [2,3,4,5]. They can impact outdoor air quality, as well as indoor air quality (IAQ). IAQ is an important field of study in recent decades, since a report by the US government’s Environmental Protection Agency (EPA) published in 1989 [6] highlighted that most people spend more than 90% of their time indoors (home, office, school, etc.). Some VOCs can have a significant impact on IAQ and human health. Indeed, a link has been shown between the presence of some VOCs and symptoms experienced by individuals, such as nasal, eye, or throat dryness and irritation, and even headaches [7]. These symptoms have been recognized and identified as sick-building syndrome (SBS) by the international community [8,9]. Besides causing SBS, some VOCs have been classified by the WHO as carcinogenic, genotoxic, and reprotoxic molecules, such as formaldehyde [10].

VOCs present in indoor air come from various sources. Some VOCs are released from outdoor emitters, such as benzene, which comes notably from urban traffic exhaust fumes [11,12], or toluene, which may be emitted in industrial activity areas [13]. VOCs are also emitted from indoor sources: building and furnishing materials, such as paint, flooring, or wood-based boards, are among the most significant emitters [14,15,16,17,18,19].

Industrialized wood-based panels such as plywood, medium-density fiberboard, particleboard, and oriented strand board are made from various types of wood, usually softwood, bonded with thermosetting adhesive resins, such as urea–formaldehyde, melamine–urea–formaldehyde, phenol–formaldehyde resins, and isocyanates such as polymeric 4,4′-diphenylmethane diisocyanate [20]. This type of panel has been shown to emit VOCs over months or even years [21,22] due to several factors such as wood species, binder levels, or type of binder [23], releasing notably carbonyl compounds (formaldehyde, benzaldehyde, hexanal) and terpenoid compounds (α- and β-pinene, camphene, limonene) [21,22].

To limit VOC emissions from boards, panels without binders can be manufactured. Many raw materials have been tested, from wood [24] to other lignocellulosic materials [25,26,27,28]. However, the majority of studies on binderless boards have focused on the pressing parameters to apply to optimize the characteristics of the boards. There is a lack of data on the emissions of VOCs from this type of board. To our knowledge, only one study has published results on emissions from binderless boards made from coriander cake [29], and has identified acetaldehyde and terpenoids as the main VOCs emitted.

Regarding the determination of VOC emissions from materials, several methods are currently used, including microchambers [30], emission test chambers [31], midget on-site emission cell (MOSEC) [32], and field and laboratory emission cell (FLEC) [33]. Most often, VOCs are preconcentrated using a sorbent cartridge or a solid-phase microextraction (SPME) fiber. VOCs are then thermodesorbed and analyzed by gas chromatography/mass spectrometry and/or flame ionization detection [34,35,36]. For carbonyl compounds, a derivatization by 2,4-dinitrophenylhydrazine (DNPH) on cartridges followed by HPLC-UV analysis is usually conducted [29,37]. The experimental measurements are commonly realized under specific test conditions [17,38]. To study VOCs, pyrolysis of materials followed by gas chromatography can also be carried out. Pyrolysis can be conducted at different temperatures, under an inert or oxidative environment, which provides a characterization of the material studied and thus of the VOCs present in the material [39,40,41].

This work aims to provide knowledge on the emissions of binderless boards made from wood. Two complementary approaches were implemented to highlight such VOC emissions: dynamic headspace extraction of VOCs using microchambers to study some targeted compounds and pyrolysis of materials to expand the range of VOCs studied. For this purpose, binderless boards were made from compression-cooking hardwood (oak) under different conditions. This is the first study of VOC emissions from such boards to our knowledge.

## 2. Materials and Methods

### 2.1. Material and Preparation

#### 2.1.1. Raw Material

The raw material was the heartwood of pedunculate oak (*Quercus robur*) harvested in the southwest of France, which was a byproduct of the industry of wine barrels.

**Figure 1:**
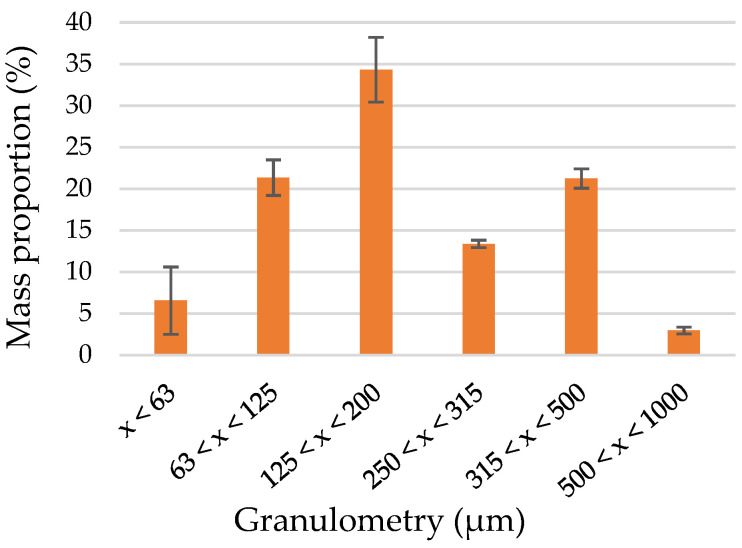
Particle size distribution of the heartwood.

The raw material was ground to 2 mm with an Electra Goulu (Moulias, France) hammer mill, and then to 1 mm with an Electra F3 (Moulias, France) hammer mill. Particle size distribution was analyzed with a Retsch AS 200 (Haan, Germany) sieving machine and six preweighted sieves (1 mm, 500 µm, 315 µm, 250 µm, 125 µm, and 63 µm). A 100 g amount of the raw materials was placed on the first sieve (1 mm), and the sieving machine was set at 75% of its amplitude for 30 min. Sieves were reweighted, and material weight retained in each sieve was obtained. The different particle sizes were expressed as a percentage. The particle size distribution of the ground heartwood is represented in Figure 1 and revealed that more than 90% of the material was found in the range of 63–500 µm, with one-third of the heartwood between 125 and 200 µm.

**Figure 2:**
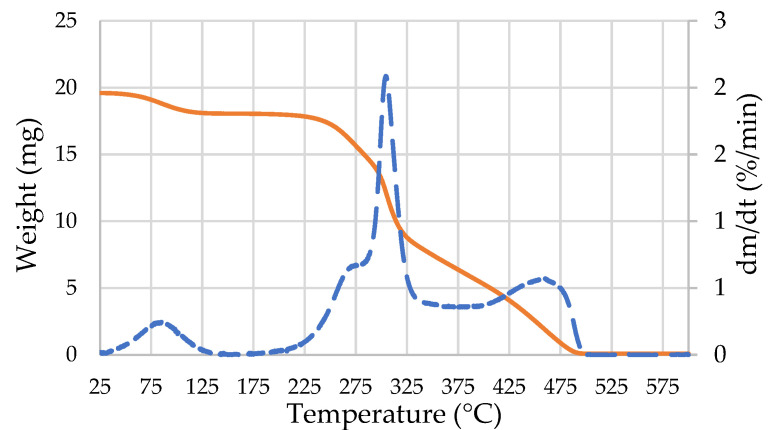
TGA (orange line) and DTG (blue dotted line) curves from the heartwood degradation.

A thermogravimetric analysis (TGA) was conducted on the raw material in order to determine the temperature at which the wood components degrade and therefore the maximum temperature not to be exceeded for the compression-cooking process. About 20 mg of the heartwood powder was placed in a 70 µL alumina crucible and analyzed on a Mettler-Toledo thermogravimetric analyzer (Columbus, OH, USA). The material was weighed from 25 °C to 600 °C at 5 °C·min^−1^, under 20 mL·min^−1^ airflow. The time derivative of the TGA curves (DTG) was calculated. The TGA curve and time derivative of the TGA curve (DTG) of the heartwood are presented in Figure 2. Three peaks can be observed on the DTG curves. The first peak is between 25 °C and 150 °C, whose maximum at 85 °C corresponds to the evaporation of water. The second peak in the range of temperature 175–350 °C can be attributed to the degradation of holocellulose [42,43], with a shoulder at 270 °C, which indicates the degradation of hemicellulose [43,44,45]. The cellulose degradation occurs during this second peak with its maximum at 304 °C [46,47,48]. Decomposition of lignin starts around 200 °C and reaches the maximum during the third peak, at 460 °C [42,48,49].

#### 2.1.2. Compression-Cooking Process

A 112.5 g amount of heartwood at 12% moisture content was thermopressed with a 400-ton capacity Pinette Emidecau Industries (Chalon-sur Saône, France) heated hydraulic press (Figure S1) without any adhesive to obtain a binderless board (15 × 15 cm) with a density of approximately 1 (Figure S2). The compression-cooking process conditions (A, B, and C) are reported in Table 1. Boards obtained with conditions A, B, and C are hereafter designated “Board A”, “Board B”, and “Board C”, respectively.

### 2.2. Reagents and Sorbents

Acetonitrile, methanol, and toluene (purity higher than 99.9%) were purchased from VWR (Radnor, PA, USA). Commercial hydrazone standard mixing solution (CARB Carbonyl-DNPH Mix 1) supplied by Sigma-Aldrich (St. Louis, MO, USA) was used as standard solution to study formaldehyde, acetaldehyde, acrolein, acetone, propanal, butanal, and benzaldehyde. A standard mixture of 20 *n*-alkanes (from C_5_ to C_40_) for retention index determination was obtained from Agilent (Santa Clara, CA, USA).

VOCs were collected in Tenax TA^®^ cartridges (300 mg, 60–80 mesh, TERA environment, Crolles, France). Carbonyl compounds were sampled on 2,4-dinitrophenylhydrazine (DNPH) silica gel cartridges (350 mg) supplied by Supelco (Bellefonte, PA, USA).

### 2.3. Evaluation of VOC Emissions

#### 2.3.1. Gaseous VOC Sampling

A microchamber/thermal extractor µ-CTE250-series (Markes International Ltd., Llantrisant, UK) with four chambers (36 mm deep, 64 mm in diameter) was used to study emissions from the heartwood and the binderless boards (Figure S3). The heartwood (1 g) was placed in aluminum cups in the microchambers. The panels were cut with a hole saw to the same diameter as the chambers to measure only the surface emissions (approximately 15 g) and were placed directly into them. Materials were left to equilibrate for 18 h before sampling. Samplings were made in accordance with the ISO 16000-9:2006 [50]. The environmental conditions in the chamber were set at 23 (±1) °C and 50 (±3)% HR. The airflow in the microchambers was set at 80 mL·min^−1^.

VOCs were collected in sorbent tubes packed with Tenax TA^®^ for 20 min at 80 mL·min^−1^. They were then thermo-desorbed and analyzed by gas chromatography and flame ionization detection (GC-FID) or mass spectrometry (GC-MS). Carbonyl compounds were trapped in DNPH silica gel cartridges, where they were derivatizated by the 2,4-dinitrophenylhydrazine for 150 min at 80 mL·min^−1^. After sampling, DNPH silica gel cartridges were eluted with 5 mL of acetonitrile to extract carbonyl compounds derivatives in acetonitrile solutions in order to identify them by HPLC-DAD.

#### 2.3.2. TD-GC-FID/MS

To identify VOCs, Tenax TA^®^ cartridges were desorbed for 10 min at 250 °C by a Perkin Elmer TurboMatrix TD (Waltham, MA, USA). The desorption flow was set at 40 mL·min^−1^. VOCs were then analyzed by a gas chromatograph Perkin Elmer Clarus 500 (PerkinElmer, Waltham, MA, USA) equipped with a quadrupole mass spectrometer Perkin Elmer Clarus 500 (Waltham, MA, USA). Compounds were separated on a DB-5ms (30 m × 0.25 mm, 0.25 μm; Agilent J&W, Santa Clara, CA, USA), with helium as carrier gas at 1 mL·min^−1^. Column temperature was held at 40 °C for 10 min and increased to 220 °C for 10 min at 6 °C·min^−1^. The detector was set at 200 °C and scanned over a mass range ratio of m/z 35–400 amu.

To quantify VOCs, VOCs were desorbed from Tenax TA^®^ tubes by a Perkin Elmer TurboMatrix 350 ATD (Waltham, MA, USA) and analyzed by a gas chromatograph Perkin Elmer AutoSystem XL (Waltham, MA, USA) equipped with a flame ionization detector (FID). The parameters of thermodesorption and separation were the same as for the TD-GC-MS, except for the column program temperature which started at 50 °C for 10 min and increased to 220 °C for 10 min at 6 °C·min^−1^. The FID was set at 250 °C, with an air flow at 430 mL·min^−1^ and 45 mL·min^−1^ for H_2_.

Identification of VOCs by GC-MS was achieved by comparison of mass spectra of compounds detected with those reported in the literature and the National Institute of Standards library (NIST, Version 2.2). A mixture of linear alkanes (C_5_ to C_40_) was injected with the same analytical parameters in order to complete the identification of compounds with their retention indices in both GC-FID and GC-MS.

Total Volatile Organic Compounds (TVOCs) are defined as the sum of the concentration of the volatile organic compounds eluting between and including *n*-hexane (C_6_) and *n*-hexadecane (C_16_). TVOCs were quantified as toluene equivalents. For this purpose, a calibration curve of toluene was established. Area-specific emission rates (SERs) are defined as the mass of a VOC or various VOCs emitted from a product per time and per exposed surface of this product. SERs were calculated following Equation (1) (according to ISO 16000-9:2006 [50]).

(1) $$\mathrm{SER}=\frac{m-m_{0}}{V}\cdot \frac{q}{S},$$

SER, area-specific emission rate (μg·m^−2^·h^−1^);
m, mass of VOC in the sorbent tube (μg);
m_0_, reference value of the glass cell (μg);
V, sampled volume (m^3^);
Q; air flow (m^3^·h^−1^);
S, sample surface (m^2^).

For the emissions of the heartwood, SER refers to the mass of heartwood, the sample surface S in Equation (1) being replaced by the mass (in grams) of the tested heartwood.

#### 2.3.3. Pyr-GC-MS

Heartwood and the three panels were ground to 0.5 mm and 2 mg were placed into pyrolysis tubes. A Pyroprobe 6150 from CDS Analytical (Oxford, PA, USA) was used as a pyrolyzer, in conjunction with a Thermo Scientific (Waltham, MA, USA) Trace 1310 model gas chromatography apparatus equipped with a Thermo Scientific TSQ 9000 mass spectrometer (Waltham, MA, USA). Samples were placed in the pyrolyzer and pyrolysis experiments were carried out at 300 °C for 30 s. Separation of the pyrolysis products was achieved using a fused-silica capillary column: TraceGOLD TG-5SilMS (30 m, 0.25 mm, 0.25 µm) from Thermo Fisher Scientific (Waltham, MA, USA). The column temperature program was set as follows: 40 °C for 2 min to 300 °C for 5 min at 10 °C·min^−1^. General profiles for pyrolyzates were obtained using EI-MS. The quadrupole was set at 300 °C and scanned over a mass range ratio of m/z 35–500 amu.

Identification was performed on peaks with an area-to-mass ratio of pyrolyzed material greater than 10^7^. Then, for the selected peaks, the identification was performed by comparison of the mass spectra with those of the compounds reported in the National Institute of Standards (NIST) library: NIST/EPA/NIH Mass Spectral Library (NIST 17) and NIST Mass Spectral Search Program (Version 2.3), for match and reverse match factors greater than 800. Relative quantification was performed by calculating each peak selected according to the previously described criterion of the relative area.

#### 2.3.4. HPLC-DAD

The acetonitrile solutions containing the carbonyl compounds collected with the DNPH silica gel cartridges were analyzed on an Ultimate 3000 high-performance liquid chromatography system (UHPLC+, ThermoFisher Scientific, Waltham, MA, USA) equipped with a diode array detector (DAD) at 350 nm. A 20 µL volume of the solution was injected, and compounds were separated through a Phenomenex (Torrance, CA, USA) Luna^®^ 5 µm C18(2) column (100 Å, 250 × 4.6 mm), thermostated at 20 °C. A mixture of water and acetonitrile at 1.3 mL·min^−1^ was used as the mobile phase with the following gradient (%acetonitrile): 0 min (30%), 15 min (50%), 26 min (50%), 33 min (70%), 36 min (95%), 40 min (95%), 42 min (30%), 45 min (30%). Carbonyl compounds were identified by comparison with a mixture of standards (CARB Carbonyl-DNPH Mix 1) containing 7 compounds: formaldehyde-2,4-DNPH, acetaldehyde-2,4-DNPH, acrolein-2,4-DNPH, acetone-2,4-DNPH, propanal-2,4-DNPH, butanal-2,4-DNPH, and benzaldehyde-2,4-DNPH. Quantification of the identified derivatives was performed using a calibration curve of the standard of each identified compound. Equation (1) was used to calculate the SERs of the hydrazones, which were then adjusted to the mass of each nonderivative compound.

#### 2.3.5. Quality Assurance

The air introduced into the microchambers was filtered through a charcoal filter to trap upstream any VOCs that might be present in the air supply, before being humidified. To check for VOCs in the background of the microchambers, air samples from the empty microchambers were collected before the materials were placed, when temperature, humidity, and airflow in the chamber had stabilized. The sampled volumes were chosen in order not to exceed the breakthrough volume of each compound [51]. Each material (heartwood or binderless board) was studied in duplicate, microchamber sampling was performed in duplicate too, and HPLC analyses were performed twice. For HPLC analyses, the quantification was carried out with calibration curves made from the standard mixture of 7 compounds mentioned above. The limit of detection (LOD) and limit of quantification (LOQ) of each compound were determined (Table 2) as the mass corresponding to a signal-to-noise ratio of 3 and 10, respectively. Pyrolysis experiments were performed in duplicate on each material. Identifications by gas chromatography/mass spectrometry were achieved by comparison of mass spectra with those of compounds reported in the National Institute of Standards (NIST) library.

### 2.4. Statistical Analysis

Analysis of variance (ANOVA) was carried out on compounds detected by the pyrolysis of materials to select the most significant compounds. Data were analyzed by principal component analysis (PCA). The relationship between compounds was studied with the Pearson coefficient. All statistical analyses were conducted with RStudio: Integrated Development for R v2022.07.1.554 (RStudio Team, PBC, Boston, USA, http://www.rstudio.com/) (accessed on 20 June 2022) and R: A Language and Environment for Statistical Computing 4.2.1 2022-06-23 (R Core Team, Vienna, Austria, https://www.r-project.org/) (accessed on 20 June 2022).

## 3. Results and Discussion

### 3.1. Hypothesis of Identification of Emitted Compounds

#### 3.1.1. Gas Chromatography Analysis

A total of 35 compounds have been identified by thermal extraction methods involving Tenax TA^®^ cartridges desorption and heartwood pyrolysis: 5 linear aldehydes, 18 aromatics, 5 furans and furan derivatives, 1 lactone, 2 lipids, 4 other identified compounds, and 11 nonidentified compounds.

Acetic acid and furfural were the major emitted compounds by the heartwood and by the binderless boards detected by GC-FID (Table 3). The main VOCs emitted by hardwoods and especially common oak are carbonyl compounds (aldehydes and carboxylic acids) and alcohols [52]. The emissions of oak are dominated by acetic acid which is produced by the hydrolysis of acetyl groups in hemicelluloses [53]. Furfural is present in the emissions of native wood, and emissions increase with the thermal treatment of wood, such as drying, due to the degradation of polysaccharides [54,55].

**Figure 3:**
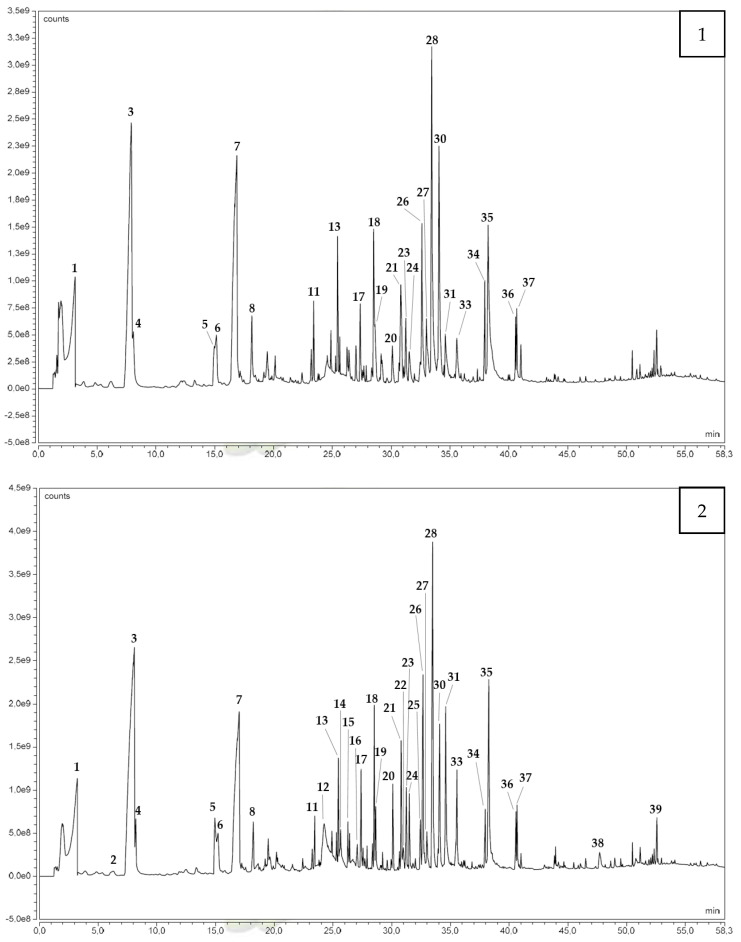
Chromatograms obtained by GC-MS related to the pyrolysis of heartwood (**1**) and related to the pyrolysis of Board A (**2**). Compounds are expressed with numbers corresponding to Table 3.

A total of 21 compounds have been identified by the pyrolysis of the heartwood (Figure 3): 14 aromatics, 3 furans and derivatives, 2 lipids, and 2 other compounds (Table 3). Acetic acid and furfural were detected after the pyrolysis of heartwood as degradation products of hemicellulose and cellulose during the pyrolysis [56]. Among the 14 aromatic compounds identified, coniferaldehyde, syringaldehyde, vanillin, and isoeugenol can be mentioned. These compounds are lignin monomers or derivatives and thus can be related to the degradation of the lignin [56]. Eight of these aromatics compounds (i.e., 4-allyl-2,6-dimethoxyphenol, 2-methoxy-4-propylphenol, 2-methoxy-4-vinylphenol, guaiacylacetone, isoeugenol, sinapaldehyde, syringaldehyde, and vanillin) were also identified, as well as acetic acid, in the study of Zhou et al., in which red oak lignin was pyrolyzed at 500 °C [57].

Choi et al. studied the oil obtained from the pyrolysis at 500 °C of red oak and identified 52 compounds by various analytical methods [58]. Among these 52 compounds, 10 were identified in this study: 2 furan derivatives (furfural and 5-methylfurfural), 7 aromatics (such as 2-methoxy-4-vinylphenol, 2,6-dimethoxy-4-vinylphenol. or 4-allyl-2,6-dimethoxyphenol), and acetic acid. A study on the pyrolysis at 450 °C of the heartwood of Quercus petraea led to the identification of 38 compounds [59], 6 of which were also identified in this study. These include furfural and acetic acid, as well as 4 aromatics: 4-allyl-2,6-dimethoxyphenol, 4-propenyl-2,6-dimethoxyphenol, syringaldehyde, and vanillin. Vichi et al. studied the composition of oak wood chips by toasting and extracting wood chips by ASE and identifying compounds by gas chromatography/mass spectrometry [60]. A total of 76 compounds were identified, 14 of which have been found here by the pyrolysis of the heartwood. These compounds include acetic acid, furfural, palmitic acid, vanillin, isoeugenol, coniferaldehyde, and sinapaldehyde.

Pyrolysis of Boards A, B, and C (Figure 3) followed by gas chromatography/mass spectrometry resulted in the identification of 28 compounds across all boards: 24 compounds for Board A, 28 compounds for Board B, and 27 compounds for Board C (Table 3). For Board A, the identified compounds were 15 aromatics, 4 furans or derivatives, 2 lipids, and 3 other compounds. The 28 compounds identified in Board B were 17 aromatics, 5 furans or derivatives, 1 lactone, 2 lipids, and 3 other compounds. Finally, by the pyrolysis of Board C, 17 aromatics, 5 furans or derivatives, 2 lipids, and 3 other compounds were identified. Twenty-four compounds have been identified in common in the three boards, most of which were also identified in the pyrolysis of the raw material (heartwood), except for syringol, 5-hydroxymethylfurfural, and lignostilbene.

From these 28 compounds identified by the pyrolysis of the boards, 21 of them have already been identified in the pyrolysis of oak at 450 °C or 500 °C [41,57,58,60]: acetic acid, furfural, 5-methylfurfural, 5-hydroxymethylfurfural (5-HMF), acetosyringone, butyrovanillone, coniferaldehyde, guaiacylacetone, isoeugenol, propiosyringone, 2-methoxy-4-propylphenol, 2-methoxy-4-vinylphenol, 2,6-dimethoxy-4-vinylphenol, 4-allyl-2,6-dimethoxyphenol, 4-propenyl-2,6-dimethoxyphenol, sinapaldehyde, sinapic alcohol, syringaldehyde, syringol, vanillin, cis-oak lactone, and palmitic acid. Omrani et al. [61] studied VOC emissions from *Quercus robur* during the wood welding process, in which the wood is heated to about 230 °C. They identified 43 compounds, 13 of which were identified here in the emissions of binderless boards (including acetic acid, furfural, 2-methoxy-4-vinylphenol, oak lactone, syringol, isoeugenol, vanillin, 2-methoxy-4-propylphenol, guaiacylacetone, acetosyringone, coniferaldehyde, syringylacetone, and sinapaldehyde), originating from the thermal degradation from either the hemicellulose or the lignin.

Since the transformation of the raw material into panels was performed at lower temperatures than the pyrolysis, most of the compounds identified during the pyrolysis of the panels are identical to those identified during the pyrolysis of the raw material.

#### 3.1.2. Liquid Chromatography Analysis

Six compounds have been identified by HPLC-DAD in the emissions of the heartwood: acetaldehyde, acrolein, benzaldehyde, butanal, formaldehyde, and propanal (Table 3). Formaldehyde is a well-known VOC emitted by wood, originating from the main components of wood (cellulose, hemicelluloses, lignin, and extractives) [65,66]. Formaldehyde, acetaldehyde, benzaldehyde, butanal, and propanal have been found in oak emissions [67], where they represent the main VOCs emitted. As for acrolein, it has been detected in the emissions from red oak (*Quercus rubra*) [68].

In the emissions of the binderless boards, acetaldehyde, acrolein, benzaldehyde, butanal, formaldehyde, and propanal have been identified in the emissions of Board C. These compounds were also identified in the emissions of Boards A and B, in addition to acetone.

### 3.2. Compression-Cooking Parameters and VOCs

#### 3.2.1. Area-Specific Emission Rates of Carbonyl Compounds and TVOCs

Area-specific emission rates (SERs) of formaldehyde, acetaldehyde, acrolein, acetone, propanal, butanal, benzaldehyde, acetic acid, furfural, total carbonyl compounds (TCCs) studied by HPLC-DAD and total volatile organic compounds (TVOCs) for the raw material and the binderless boards are reported in Table 4. The main carbonyl compound emitted by the binderless boards was acetaldehyde, with SERs of 781 µg·m^−2^·h^−1^, 87 µg·m^−2^·h^−1^, and 109 µg·m^−2^·h^−1^ for Boards A, B, and C, respectively, while acrolein was the major carbonyl compound emitted by the raw material (1080 ng·g^−1^·h^−1^). Emissions of formaldehyde from binderless boards varied from 31 to 61 µg·m^−2^·h^−1^ compared to 30-840 µg·m^−2^·h^−1^ for industrialized boards (medium-density fiberboard, hardboard, or plywood) under the same conditions of temperature and humidity [18,69,70]. For all compounds studied by HPLC-DAD (formaldehyde, acetaldehyde, acrolein, acetone, propanal, and butanal), Board A was found to be the largest emitter of the three binderless boards, followed by Board C and then Board B. Indeed, the SER of TCCs was 1060 µg·m^−2^·h^−1^ for Board A, against 183 µg·m^−2^·h^−1^ for Board C and 149 µg·m^−2^·h^−1^ for Board B. Benzaldehyde was identified in the emissions of all three boards and of the raw material, but was not quantified, as it was below the limit of quantification (LOQSER). 

Regarding the compounds analyzed by gas chromatography (acetic acid and furfural), the results were similar to the compounds studied in liquid chromatography. In fact, Board A was found to be the most important emitter of acetic acid, furfural, and TVOCs, with SERs of 386 µg_eq toluene_·m^−2^·h^−1^, 336 µg_eq toluene_·m^−2^·h^−1^, and 837 µg_eq toluene_·m^−2^·h^−1^, respectively. Board B emitted less acetic acid and furfural than the other binderless boards (respectively, 84 µg_eq toluene_·m^−2^·h^−1^ and 26 µg_eq toluene_·m^−2^·h^−1^), and thus the SER of TVOCs was the lowest among the binderless boards (110 µg_eq toluene_·m^−2^·h^−1^). Emissions of TVOCs from industrial boards bounded with petrochemical adhesives have been studied and found to vary under the same conditions of temperature and humidity between 30 and 3100 µg_eq toluene_·m^−2^·h^−1^ [69,71,72] depending on the type of board, the thickness, or the wood species.

Comparing the emissions of these 9 compounds, TCCs, and TVOCs, Board A, which was manufactured at a high temperature (170 °C) and long time (6 min), was found to be the most emitting board. On the contrary, Board B, which was thermopressed with the least severe conditions (129 °C, 1 min), emitted less than the other binderless boards. Increasing pressing temperature and pressing time resulted in an increase in the emissions for the studied compounds. Xue et al. [73] also observed an increase in the aldehyde emissions from poplar air-dried at 160 °C to 180 °C and especially of furfural and acetic acid emissions. The same observation was made by Manninen et al. [74], who compared the VOC emissions between air-dried and heat-treated Scots pine wood.

#### 3.2.2. Pyrolysis as a Tool to Evaluate Wood-Panel Released Compounds

**Figure 4:**
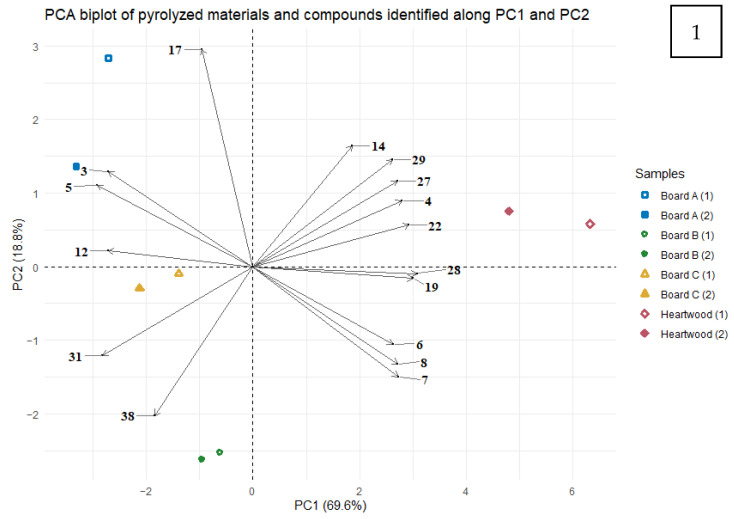

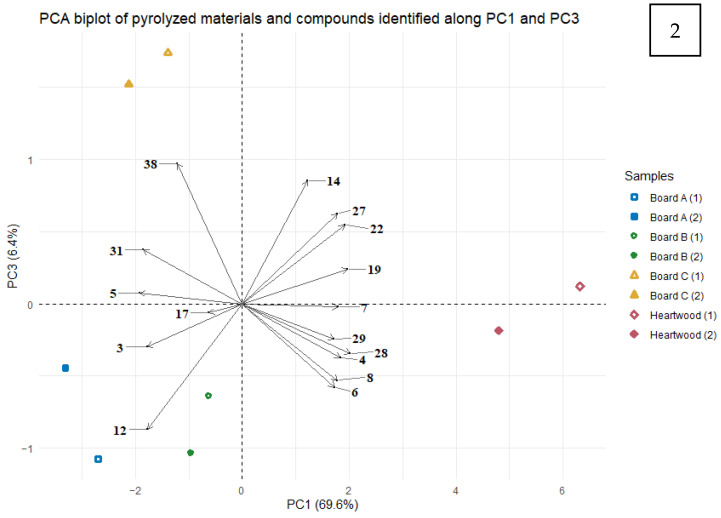
PCA biplot of individuals (samples) and variables (compounds identified by pyrolysis–GC-MS) along PC1 and PC2 dimensions (**1**) and along PC1 and PC3 dimensions (**2**). Variables are expressed with numbers corresponding to Table 3.

Among the 39 compounds identified by pyrolysis-GC-MS, 16 were significant to discriminate between wood panels (ANOVA, *p* < 0.05), including 5 aromatic compounds, 4 furans and derivatives, 1 lactone, 1 stilbene, and 5 nonidentified compounds. Principal component analysis (PCA) on these 16 compounds (Figure 4)—first, second, and third components (PC1, PC2, and PC3)—represented 94.8% of the variance between samples, with, respectively, 69.6%, 18.8%, and 6.4% of the explained variance. Relationships between compounds (higher than 90%) were also investigated.

Among the 16 compounds, 13 were found to be the largest contributors to the variance between samples regarding PC1: furfural (**3**), 3-furaldehyde (**4**), 5-methylfurfural (**5**), 5-HMF (**12**), 2-methoxy-4-propylphenol (**19**), 4-propenyl-2,6-dimethoxyphenol (**28**), acetosyringone (**29**), syringylacetone (**31**), and the 5 nonidentified compounds (**6**, **7**, **8**, **22**, and **27**). These compounds highlighted the significant difference between the raw material and the binderless boards (*p* < 0.001). Indeed, the raw material was characterized by the highest relative contents of nine compounds, including three aromatic compounds (2-methoxy-4-propylphenol, 4-propenyl-2,6-dimethoxyphenol and acetosyringone), one furan (3-furaldehyde), and the five nonidentified compounds. The aromatic compounds were chemically modified during the manufacture of the boards, while the lignin in the raw material undergoes a heat treatment during the pyrolysis at 300 °C, leading to the release by the heartwood of monomers or monomer derivatives (guaiacyl and syringyl units), as extractives [75,76]. On the contrary, the boards presented greater relative quantities of furans and derivatives, such as furfural, 5-methylfurfural, and 5-HMF, than the heartwood. Compounds **3** and **5** (furfural and 5-methylfurfural) were positively correlated (R = 0.95). These kinds of compounds (furans and derivatives) are formed during the compression-cooking process from the depolymerization of cellulose and hemicellulose [56]. Board A, which had the most severe compression-cooking conditions (170 °C, 6 min, 30 MPa), was found to have the highest relative contents of furfural, 5-methylfurfural, and 5-HMF, while they were found in lower relative quantities in Board B, which had the lowest pressing temperature (129 °C, 6 min, 30 MPa). These three compounds and more particularly their quantities in the pyrolyzed materials thus reflected the thermopressing conditions, and especially the pressing temperature. Regarding PC2, only vanillin (**17**) was significantly correlated to the second component axis. Vanillin was found to have the highest relative content in Board A (170 °C, 6 min, 30 MPa), whereas Board B (129 °C, 6 min, 30 MPa) showed the lowest relative amount among the 3 binderless boards. Of all the compounds identified and studied by PCA, vanillin is the compound that has the highest boiling point. The relative quantification of vanillin showed a significant difference between Board B and the other materials (*p* < 0.01). Finally, Board C was found to be significantly different from the other materials regarding PC3 (*p* < 0.001), meaning that the thermopressing time has an impact on the composition of the binderless boards. Board C (170 °C, 1 min, 30 MPa) was characterized by the highest relative amount of lignostilbene (**38**). Compounds **6**, **7**, and **8** (corresponding to unidentified Compounds **2**, **3,** and **4**) behave identically. Indeed, they show positive correlations between them (R² > 0.9), and each is negatively correlated with 5-methylfurfural (**5**). Syringylacetone (**31**) is negatively correlated with 2 other aromatic compounds: acetosyringone (**28**) and syringylacetone (**29**), with coefficients of −0.90 and −0.93, respectively. As these three compounds are derivatives of syringyl units of lignin, there is competition between those compounds during the breaking of the lignin bonds.

Analyses of the pyrolyzed boards by PCA revealed that the compression-cooking temperature (129 °C or 170 °C) and time (1 min or 6 min) induced a significant difference in the composition of the boards. The PCA also highlighted the transformation of the raw material due to the process. The PCA on the relative amounts of compounds present in the pyrolyzed materials thus highlighted compounds that are transformed during the compression-cooking process and provided some pyrolysis markers of the transformation of the raw material.

## 4. Conclusions

A total of 31 VOCs were identified in boards obtained under the most severe compression-cooking conditions (A), 35 in boards obtained with the lowest pressing temperature (B), and 33 in boards obtained at the highest compression-cooking temperature but with a short processing time (C). Compounds highlighted notably included formaldehyde, acetaldehyde, furfural, coniferaldehyde, syringaldehyde, or vanillin. Pyrolysis–GC-MS data results revealed that oak hardwood presented more aromatic compounds, while the binderless boards presented more furans and derivatives. Moreover, the boards made at higher temperatures showed higher values of furans and derivatives, as well as of vanillin. Finally, using analytical pyrolysis, this study provided some characteristic compounds of the transformation of oak heartwood during the compression firing treatment.

The two complementary approaches used in the study (microchamber extraction and analytical pyrolysis) can serve as a basis to further study the impact of compression-cooking parameters on the VOCs from materials. The ultimate goal of these studies is focused on reducing the VOC content of the processed boards.

## Figures and Tables

**Table 1:** Compression-cooking process conditions.

Pressing Parameters	Conditions A	Conditions B	Conditions C
Pressing temperature (°C)	170	129	170
Pressing time (min)	6	6	1
Pressing pressure (MPa)	30	30	30

**Table 2:** LOD and LOQ of quantified compounds.

Compound	LOD(ng)	LOQ(ng)	LODSER ^(a)^		LOQSER ^(a)^	
			(µg·m^−2^·h^−1^)	(ng·g_material_^−1^·h^−1^)	(µg·m^−2^·h^−1^)	(ng·g_material_^−1^·h^−1^)
Formaldehyde	0.7	1.0	0.1	0.3	0.2	0.4
Acetaldehyde	0.9	1.2	0.1	0.4	0.2	0.5
Acrolein	1.0	1.7	0.1	0.4	0.2	0.7
Acetone	1.0	1.6	0.1	0.4	0.2	0.7
Propanal	0.8	1.3	0.1	0.3	0.2	0.5
Butanal	0.9	1.3	0.1	0.4	0.2	0.5
Benzaldehyde	2.1	3.6	0.3	0.8	0.5	1.5
Toluene	0.6	1.2	0.6	1.8	1.2	3.7

^(a)^ For a sampled volume of 12 L on DNPH silica gel cartridges, except for toluene (sampled volume of 1.6 L on Tenax TA^®^ cartridges).

**Table 3:** List of tentatively identified compounds by thermal extraction of the different materials studied.

Compound Family	No.	Compounds	Chemical Formula	CAS	RI_th_ (DB-5MS-Like)	Boiling Points (°C) [62]	Identification Method			
							Heartwood	Board A	Board B	Board C
Aldehydes (linear)	**41**	Acetaldehyde	C_2_H_4_O	75-07-0		21	LC	LC	LC	LC
	**42**	Acrolein	C_3_H_4_O	107-02-8		52	LC	LC	LC	LC
	**44**	Butanal	C_4_H_8_O	123-72-8		75	LC	LC	LC	LC
	**40**	Formaldehyde	CH_2_O	50-00-0		-19	LC	LC	LC	LC
	**43**	Propanal	C_3_H_6_O	123-38-6		48	LC	LC	LC	LC
Aromatics	**29**	Acetosyringone	C_10_H_12_O_4_	2478-38-8	1739 ^(b)^				Pyr	Pyr
	**24**	4-Allyl-2,6-dimethoxyphenol	C_11_H_14_O_3_	6627-88-9	1608 ^(b)^		Pyr	Pyr	Pyr	Pyr
	**45**	Benzaldehyde	C_7_H_6_O	100-52-7		179	LC	LC	LC	LC
	**23**	Butyrovanillone	C_11_H_14_O_3_	64142-23-0	1593 ^(b)^		Pyr	Pyr	Pyr	Pyr
	**30**	Coniferaldehyde	C_10_H_10_O_3_	458-36-6	1728 ^(a)^		Pyr	Pyr	Pyr	Pyr
	**21**	2,6-Dimethoxy-4-vinylphenol	C_10_H_12_O_3_	28343-22-8	1573 ^(c)^		Pyr	Pyr	Pyr	Pyr
	**20**	Guaiacylacetone	C_10_H_12_O_3_	2503-46-0	1571 ^(b)^		Pyr	Pyr	Pyr	Pyr
	**18**	Isoeugenol	C_10_H_12_O_2_	97-54-1	1448 ^(a)^	264	Pyr	Pyr	Pyr	Pyr
	**19**	2-Methoxy-4-propylphenol	C_10_H_14_O_2_	2785-87-7	1366 ^(a)^	121	Pyr	Pyr	Pyr	Pyr
	**13**	2-Methoxy-4-vinylphenol	C_9_H_10_O_2_	7786-61-0	1309 ^(a)^		Pyr	Pyr	Pyr	Pyr
	**28**	4-Propenyl-2,6-dimethoxyphenol	C_11_H_14_O_3_	20675-95-0	1704 ^(c)^		Pyr	Pyr	Pyr	Pyr
	**33**	Propiosyringone	C_11_H_14_O_4_	5650-43-1	1827 ^(b)^		Pyr	Pyr	Pyr	Pyr
	**32**	Sinapyl alcohol	C_11_H_14_O_4_	537-33-7	1998 ^(b)^				Pyr	Pyr
	**35**	Sinapaldehyde	C_11_H_12_O_4_	4206-58-0	1989 ^(b)^		Pyr	Pyr	Pyr	Pyr
	**26**	Syringaldehyde	C_9_H_19_O_4_	134-96-3	1655 ^(a)^	192	Pyr	Pyr	Pyr	Pyr
	**31**	Syringylacetone	C_12_H_16_O_4_	112468-41-4	1746 ^(c)^		Pyr	Pyr	Pyr	Pyr
	**15**	Syringol	C_8_H_10_O_3_	91-10-1	1346 ^(a)^	261		Pyr	Pyr	Pyr
	**17**	Vanillin	C_8_H_8_O_3_	121-33-5	1393 ^(a)^	285	Pyr	Pyr	Pyr	Pyr
Furans and derivatives	**4**	3-Furaldehyde	C_5_H_4_O_2_	498-60-2	832 ^(c)^	145	Pyr	Pyr	Pyr	Pyr
	**3**	Furfural	C_5_H_4_O_2_	98-01-1	828 ^(a)^	162	Pyr, Th	Pyr, Th	Pyr, Th	Pyr, Th
	**9**	4-Hydroxy-5-methylfuran-3(2H)-one	C_5_H_6_O_3_	19322-27-1	1042 ^(c)^				Pyr	Pyr
	**12**	5-Hydroxymethylfurfural	C_6_H_6_O_3_	67-47-0	1215 ^(b)^	115		Pyr	Pyr	Pyr
	**5**	5-Methylfurfural	C_6_H_6_O_2_	620-02-0	957 ^(a)^	186	Pyr, Th	Pyr, Th	Pyr, Th	Pyr, Th
Lactones	**14**	*cis*-Oak lactone	C_9_H_16_O_2_	55013-32-6	1327 ^(b)^				Pyr	
Lipids	**37**	Oleic acid	C_18_H_34_O_2_	112-80-1	2142 ^(a)^	360	Pyr	Pyr	Pyr	Pyr
	**34**	Palmitic acid	C_16_H_32_O_2_	57-10-3	1959 ^(a)^	351	Pyr	Pyr	Pyr	Pyr
Others	**1**	Acetic acid	C_2_H_4_O_2_	64-19-7	606 ^(a)^	118	Pyr, Th	Pyr, Th	Pyr, Th	Pyr, Th
	**43**	Acetone	C_3_H_6_O	67-64-1		56		LC	LC	
	**38**	Lignostilbene	C_16_H_16_O_4_	7329-69-3				Pyr	Pyr	Pyr
	**11**	6-Propyl-5,6-dihydro-2H-pyran-2-one	C_8_H_12_O_2_	16400-69-4	1268 ^(c)^		Pyr	Pyr	Pyr	Pyr
	**2**	N.I. 1 (84, 55, 85) *	-	-	-	-			Pyr	Pyr
	**6**	N.I. 2 (110, 109, 53) *	-	-	-	-	Pyr	Pyr	Pyr	Pyr
	**7**	N.I. 3 (114, 58, 57) *	-	-	-	-	Pyr	Pyr	Pyr	Pyr
	**8**	N.I. 4 (113, 123, 58) *	-	-	-	-	Pyr	Pyr	Pyr	Pyr
	**10**	N.I. 5 (128, 129, 42) *	-	-	-	-				Pyr
	**16**	N.I. 6 (43, 172, 29) *	-	-	-	-			Pyr	Pyr
	**22**	N.I. 7 (137, 124, 180) *	-	-	-	-		Pyr	Pyr	Pyr
	**25**	N.I. 8 (194, 91, 119) *	-	-	-	-		Pyr	Pyr	Pyr
	**27**	N.I. 9 (195, 131, 132) *	-	-	-	-	Pyr	Pyr	Pyr	Pyr
	**36**	N.I. 10 (81, 67, 95) *	-	-	-	-	Pyr	Pyr	Pyr	Pyr
	**39**	N.I. 11 (147, 145, 105) *	-	-	-	-		Pyr	Pyr	Pyr

N.I: non identified; RI_th_: theoretical retention indices given by ^(a)^ Adams (2007) [63], ^(b)^ Vichi et al. (2007) [60], and ^(c)^ NIST WebBook [64]; LC: HPLC-DAD; Pyr: Pyr-GC-MS; Th: TD-GC-FID/MS; *: m/z and % of the three most intense peaks for N.I. compounds, m/z corresponding to peak base is underlined.

**Table 4:** SERs from the raw material and the binderless boards.

Compounds	Heartwood (ng·g^−1^·h^−1^)	Board A	Board B	Board C
		(µg·m^−2^·h^−1^)		
Formaldehyde	186.9 ± 9.0	60.5 ± 9.4	30.7 ± 2.0	44.2 ± 3.1
Acetaldehyde	313 ± 73	782 ± 97	87.1 ± 4.0	109.8 ± 9.8
Acrolein	(108 ± 33)·10^1^	187 ± 57	15.7 ± 4.2	25.8 ± 2.6
Acetone	<0.4 *	6.5 ± 1.2	4.1 ± 0.6	<0.2 *
Propanal	13.4 ± 1.1	11.0 ± 2.7	4.8 ± 1.2	2.1 ± 1.0
Butanal	5.1 ± 4.0	13.8 ± 3.8	7.2 ± 0.3	1.3 ± 0.7
Benzaldehyde	<1.5 **	<0.5 **	<0.5 **	<0.5 **
TCCs	(160 ± 41)·10^1^	(106 ± 17)·10^1^	150 ± 12	183 ± 17
Acetic acid	(106 ± 15)·10^4 (a)^	387 ± 68 ^(b)^	84 ± 61 ^(b)^	180 ± 29 ^(b)^
Furfural	(360 ± 50)·10^2 (a)^	337 ± 58 ^(b)^	27 ± 11 ^(b)^	126 ± 18 ^(b)^
TVOCs	(1235 ± 53)·10^3 (a)^	(838 ± 15)·10^1 (b)^	111 ± 46 ^(b)^	301 ± 50 ^(b)^

^(a)^ SER given in ng_eq toluene_·g^−1^·h^−1^; ^(b)^ SER given in µg_eq toluene_.m^−2^·h^−1^; TCCs: total carbonyl compounds (studied by HPLC-DAD); TVOCs: total volatile organic compounds; * LODSER (limit of detection for each carbonyl compound calculated for a sample volume of 12 L); ** LOQSER (limit of quantifi cation for each carbonyl compound calculated for a sample volume of 12 L).

## Data Availability

The data presented in this study are available on request to the corresponding author.

## Supplementary Materials

The following supporting information can be downloaded at: https://www.mdpi.com/article/10.3390/molecules27238260/s1, Figure S1: Hydraulic press used for this study; Figure S2: Photographs of manufactured boards; Figure S3: Microchamber system for VOC sampling.

## Sample Availability

Samples of the compounds are not available from the authors.
