# Supplementary material for: Microchamber Extraction and Analytical Pyrolysis to Explore Volatile Organic Compounds from Compression-Cooking Wood Materials Obtained under Different Conditions
Source: Molecules. 2022 Nov 26;27(23):8260. doi: 10.3390/molecules27238260 (PMC9735594; doi:10.3390/molecules27238260)
Supplement: Supplementary file 1 [file molecules-27-08260-s001.zip › molecules-1980402-supplementary.pdf]

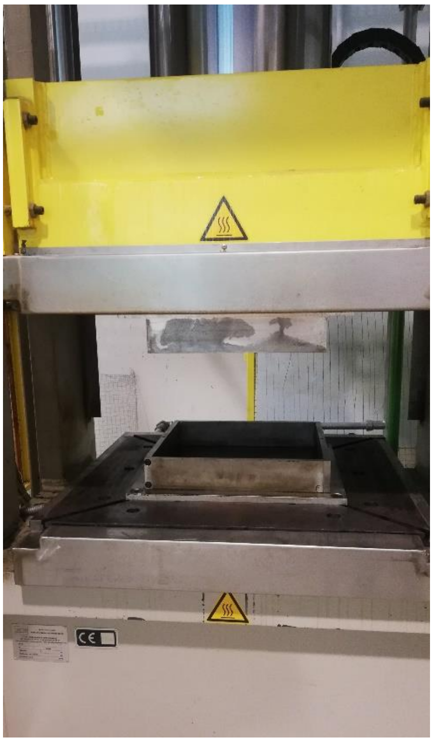

Figure S1. Hydraulic press used for this study.
(Mold: 40 cm)

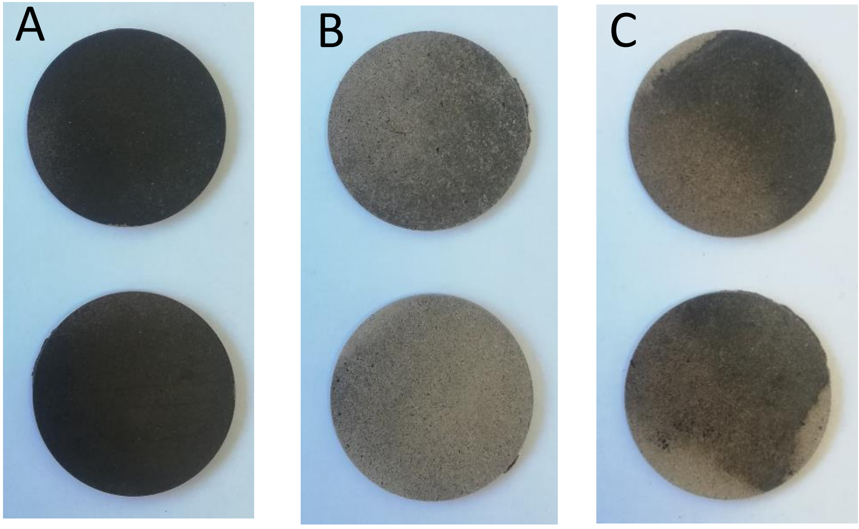

Figure S2. Photographs of manufactured boards.
(Letters corresponding to the compression-cooking conditions from Table 1, diameter: 6.4 cm)

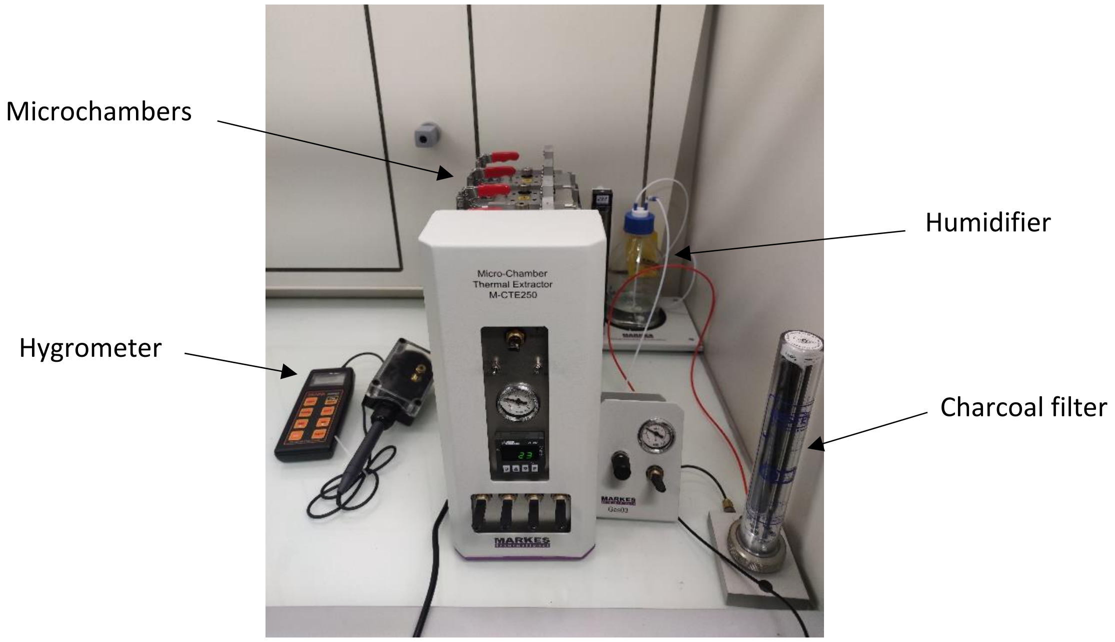

Figure S3. Microchamber system for VOC sampling.
